# Eye Care in Young Children: A Parents’ Perspective of Access and Barriers

**DOI:** 10.18502/jovr.v18i2.13186

**Published:** 2023-04-19

**Authors:** Ali M Alsaqr

**Affiliations:** ^1^Department of Optometry, College of Applied Medical Sciences, King Saud University, Saudi Arabia

**Keywords:** Amblyopia, Refractive Errors, Saudi Arabia, Strabismus, Vision Disorders, Vision Screening

## Abstract

**Purpose:**

To evaluate parental perspectives of accessing eye care for children aged under seven years.

**Methods:**

The survey was conducted during September 2020 to March 2021 using online applications and distributed to parents whose children were between the ages of three and seven years. The survey included parents' background, their knowledge of the provision of eye-care services, and the possible barriers that existed to access eye-care services. The relationship between parents' knowledge, barrier scores, level of parental education, and demographic or socioeconomic status was assessed using nonparametric tests.

**Results:**

In total, 1037 questionnaires were completed. The respondents were from 50 cities across Saudi regions. The participants' age was 39 ± 7.5 years, and 54% of them had at least one child under the age of seven (*n* = 564). Further, 47% had not taken their children for vision screening at reception/year one (*n* = 467). In addition, 65% of them were not aware of the mandatory screening program at reception/year *1*; whereas, only 20% (*n* = 207) knew how to access eye-care services; and only 39% of the children had undergone any kind of eye or vision test. The pathways to eye care and the cost of eye services/glasses were the main limitations. The parents' responses were significantly influenced by their demographic and socioeconomic characteristics (Kruskal Wallis, *P* < 0.05).

**Conclusion:**

There was a need for enhancing parent information on how to access eye care for young children and the currently available vision screening programs. Finally, a national protocol to cover the cost of the eye exam as well as spectacle prescription shall be proposed as a mean of incentive.

##  INTRODUCTION

A great number of population-based studies have indicated that the main visual disorders in children are refractive errors, amblyopia, and strabismus.^[1,2,3,4,5,6,7,8,9,10,11,12]^ Early proactive interventions for reduced vision are predominantly important during this critical period of visual development in children and should be started as early as possible.^[7,13]^ Reduced visual acuity has marked implications on education, health, social outcomes, and quality of life of affected children.^[14,15,16,17,18,19,20]^ If untreated or not detected early, these disorders would eventually lead to amblyopia and visual impairment.^[21,22]^ Furthermore, in 2007, it was estimated that uncorrected refractive errors have a global economic burden of approximately $269 billion per annum because of productivity losses.^[23]^ Specifically, several studies have stated the importance of vision screening in children under seven years old.^[10,20,24]^ These children are at risk of functionally low vision.^[25,26]^

In the agenda of VISION 2020 (The Right to Sight), the World Health Organization set the management of childhood visual disability as a priority.^[27]^ The American Academy of Pediatrics, the American Association for Pediatric Ophthalmology and Strabismus, and the American Academy of Ophthalmology have also set a joint policy statement on child vision screening.^[27]^ Preschool vision screening policies vary due to differences in the policies that exist in countries.^[13,16,17,26]^ In Saudi Arabia (SA), according to government laws, the Ministry of Education requires an obligatory medical examination, which includes an assessment of visual acuity for all school entrants.^[28]^ However, it is observed that examination facilities may be inadequate.^[11]^

Interested individuals involved in preschool vision screenings include parents, school teachers, and health professionals (optometrists, ophthalmologists, etc.). The perception, awareness, and level of accepted responsibilities of these individuals could play a crucial role in the efficacy of child vision screening programs and the development of policies for school and preschool-age children.^[24,29,30,31]^ A study conducted in England reported that approximately 30% of children did not attend follow-up visits after failing screening tests at schools' entry year.^[9]^ Several studies have emphasized the importance of parents' awareness in combating children's visual problems.^[29,30,31,32]^ The parents' knowledge of the potential visual disorders at younger age and receiving the screening outcome of children who failed visual screening could be essential for seeking health counseling.^[18,29,32]^ Specifically, parents as caregivers play the fundamental role in seeking eye-care services for their children to avoid experiencing visual disorders that may go untreated.^[33]^ Parents' socioeconomic status could also pose as an important factor when accessing eye-care services.^[8,34,35]^

To date, very few studies have been done on accessibility and barriers to eye care for children in SA, and generally in the Middle East region. The problems outlined through this research is of critical importance toward understanding the extent and complexity of the challenges facing policy makers and eye-care professionals. This knowledge gap provided an opportunity to establish a point of reference as compared to other studies conducted in other worldwide countries.^[7,31,32,36,37,38]^ Therefore, this study evaluated parents' knowledge of how to access eye care and what barriers might disable them from accessing eye care for their children.

##  METHODS

The study protocol was reviewed and approved by the IRB ethical committee of King Saud University, Saudi Arabia, and the approval number is E-22-7412. In addition, the protocol of the study complied with the guidelines for human studies and the World Medical Association Declaration of Helsinki, and parental consent was electronically obtained before filling out the questionnaires. In order to ensure transparency and to receive honest responses from participants, information and aims of the survey were absolutely and clearly described to the parents at the beginning of the survey.

This study is cross-sectional in design and targeted toward the parents of children under seven years of age in different regions of SA. The survey used in this study was adapted from a previously published study.^[32]^ The survey involved parents' demographic data, general medical and ocular history, and their knowledge and barriers regarding accessing eye-care services.

To compute the required sample size, we used Epi Info, version 7 (Centers for Disease Control, Atlanta, GA, USA; http://wwwn.cdc.gov/epiinfo/7/), and the number of children under seven years in SA were approximately 6 million.^[39]^ Furthermore, in the calculation, 95% confidence intervals, an expected frequency of 50%, a design effect of 2, and the number of clusters of five regions (central, northern, western, eastern, and southern regions) were included. The overall sample size was estimated to be 760 parents. The sample was expected to be unequal in each cluster but proportionate to the number of inhabitants in each region as they differed to a large extent (e.g., central region has approximately 8 million inhabitants and there are approximately 2 million inhabitants in the northern region). The survey was promoted for about six months (from September 2020 to March 2021) in order to recruit sufficient participants representing the Saudi population.

The survey used an online questionnaire, which was accessible without any restrictions. Emails were sent to the members of the Saudi Optometry Society to promote the survey in their areas using all accessible legal means, and the survey was distributed using all available social media applications (e.g., Twitter, WhatsApp, and Telegram). To avoid duplicate responses, at the beginning of the survey, a note was placed stating that responding to the survey more than once is prohibited. Lastly, the raw responses were properly reviewed and checked for duplication and to detect the parents who did not have children under seven years old, and eventually, 125 responses out of the 1162 initial ones were excluded.

Data were explored for normality using the Kolmogorov–Smirnov test, which indicated that the data was not normally distributed. Therefore, the nonparametric Kruskal–Wallis test was performed to consider any possible relationship among the factors of parents' knowledge, barrier scores, level of parental education, and demographic or socioeconomic status. Data were collected in Excel (Microsoft Corporation, Redmond, WA, USA) and analyzed using the Statistical Package for the Social Sciences (IBM Corp., Armonk, NY, USA).

**Figure 1:**
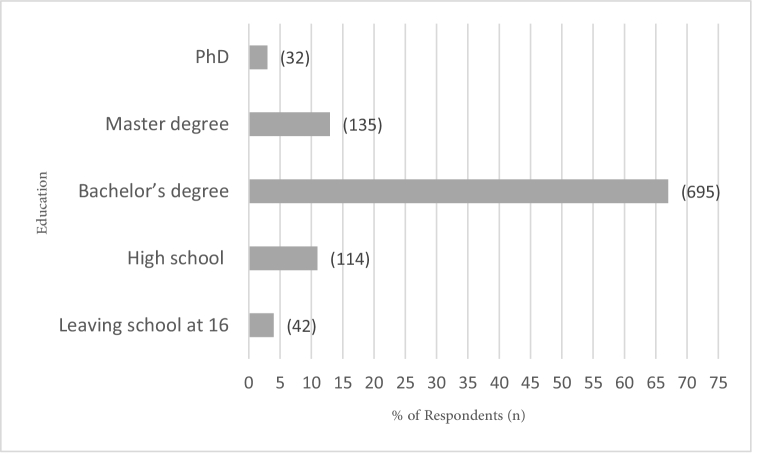
Respondents' education profiles, Saudi Arabia in year 2021.

**Figure 2:**
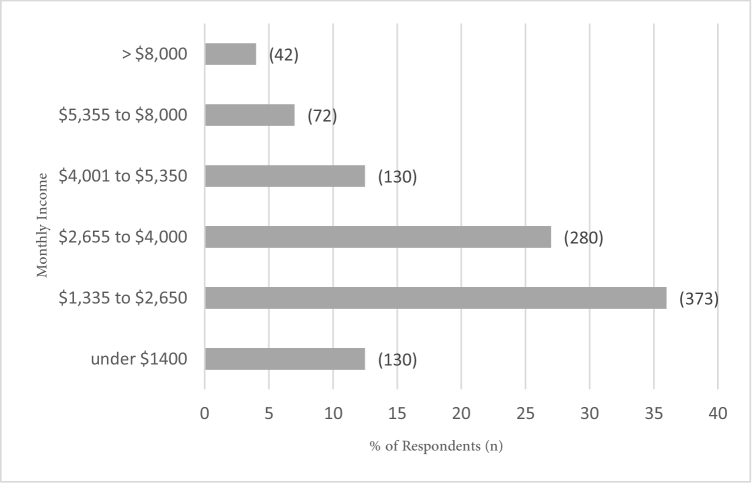
Respondents' monthly income profiles, Saudi Arabia in year 2021.

**Table 1:** Parental responses to the questions on the current visual status of their children, Saudi Arabia in year 2021.


**Query**	**Yes (** ***n*** **, %)**	**No (** ***n*** **, %)**	**Not sure (** ***n*** **, %)**
Routine vision screening in child's school	99, 9.5%	793, 76.5%	145, 14%
Concerns about child's eyes or vision	436, 42%	441, 42.5%	160, 15.5%
Know how to access an eye test appropriate for your child's age	207, 20%	778, 75%	52, 5%
Child's close family members who wear glasses and have a lazy eye or an eye turn	736, 71%	259, 25%	42, 4%
Has child ever had any kind of eye or vision test?	405, 39%	601, 58%	31, 3%
Has child ever been refused an eye test?	36, 3.5%	990, 95.5%	11, 1%
From previous question, what reason was given if an eye test was refused?	7 of 36, 19.5%	21 of 36, 58%	8 of 36, 22%
	
	

**Table 2:** Parental responses to the question stating, "For what reasons would you consider seeking an eye examination for your child?" The parents were allowed to choose more than one choice, Saudi Arabia in year 2021.


**Inquiry**	**Response (** ***n*** **,%)**
Advised by healthcare provider or teacher	347, 33.5%
Concerns about poor vision	550, 53%
Concerns about eyes not being straight/having an eye turn	249, 24%
Headaches	239, 23%
Poor concentration/short attention span	300, 29%
Poor school achievement and/or difficulties with literacy	224, 22.50%
Complaints of double vision	166, 16%
Routine checkup	353, 34%
Family history	322, 31%
Others	36, 3.50%
	
	

**Table 3:** Parental responses toward questions directed to existing knowledge about child vision and vision screening, Saudi Arabia in year 2021.


**Query**	**Agree (** ***n*** **, %)**	**Disagree (** ***n*** **, %)**	**Not sure (** ***n*** **, %)**
Children can only have an eye test when they know the names of the letters	207, 20%	498, 48%	332, 32%
Wearing glasses if you need them when under age of seven years will make your eyes and vision stronger	492, 47.5%	166, 16%	379, 36.5%
It is normal for a child aged one to seven years to occasionally have an eye turn	264, 25.5%	332, 32%	441, 42.5%
School vision screening tests for all eye problems	254, 24.5%	410, 39.5%	373, 36%
	
	

**Table 4:** Parental responses to the question stating the possible reasons that may prevent the parents from taking their children for an eye test. The parents were allowed to choose more than one choice, Saudi Arabia in year 2021.


**Inquiry**	**Response (** ***n*** **,%)**
I do not know how and/or where to arrange an appointment for an eye test	342, 33%
I am worried about the cost of an eye test	135, 13%
I am worried about the cost of glasses	82, 8%
I think my child is too young to have an eye test	270, 26%
I am worried my child does 'not know all the letters yet	124, 12%
I have been told that my child is too young for an eye test	52, 5%
I do not want my child to wear glasses	114, 11%
I am worried my child may be given glasses he/she does not need	218, 21%
I am worried if my child is given glasses that it will make his/her eyes weaker	156, 15%
Others	73, 7%
	
	

**Table 5:** Influence of the participants' background characteristics on some of the parents' responses, Saudi Arabia in year 2021.


**Factors**	**Findings**	**Kruskal–Wallis test**
Gender	The number of children who have been tested at reception/year one in mothers' response was greater than that in males.	H (2) = 5.22; *P* = 0.02
	The mothers was higher than that of males who had been refused eye-care services for their children.	H (2) = 5.73; *P* = 0.02
Marital status	Married couples were more informed about the mandatory eye exam at reception/year one.	H (2) = 6.26; *P* = 0.04
Age	Older parents were more likely to test their children at reception/year one.	H (4) = 17, *P* = 0.001
	Older parents were more informed about the mandatory eye exam at reception/year one.	H (4) = 9.6, *P* = 0.02
	Older parents were more informed about eye tests conducted at schools.	H (4) = 7.9, *P* = 0.048
	Older parents were more likely to take their young children to eye-care service.	H (4) = 18.6, *P* < 0.0001
	Older parents were more likely to have a medical eye history.	H (4) = 22.3, *P* < 0.0001
Parents' education	The higher the parents' education, the more they know about the mandatory eye examination at reception/year one.	H (4) = 12.5, *P* = 0.01
	The higher the parents' education, the more they know about pathways on how to access eye-care service.	H (4) = 15.4, *P* = 0.004
	The higher the parents' education, the more they positively believe that using glasses, if needed, under the age of seven years will make their children's vision stronger.	H (4) = 13.7, *P* = 0.01
Parents' working status	Teachers were the most informed about the mandatory eye examination at reception/year one.	H (6) = 13.8, *P* = 0.01
	Teachers had more knowledge about the routine eye examinations performed at schools.	H (6) = 11.66, *P* = 0.02
	Teachers had more concerns about their children's vision.	H (6) = 12.7, *P* = 0.01
	Housewives accounted for the greatest number of those who believe that wearing glasses, if needed, will make their children's eyes and vision stronger.	H (6) = 10.6, *P *= 0.03
	Housewives had the most number among those not knowing how to access eye-care services.	H (6) = 11.69, *P* = 0.02
Parents' income	Children of parents who had a lesser income were the least of being tested at reception/year one.	H (5) = 11.5, *P* = 0.04
	Parents with lesser income were more likely to be refused to provide eye-care services.	H (5) = 15.2, *P* = 0.01
	Parents with lesser income were more likely to believe that it is normal for a child under the age of seven years to occasionally have an eye turn.	H (5) = 18.2, *P* = 0.003
Presence of ocular history	Parents with an ocular history tend to not test their children at reception/year one.	H (2) = 6.9; *P* = 0.01
	Parents with an ocular history have the highest response of "yes" among those who have been provided with reasons for refusing eye-care services.	H (2) = 5.5; *P* = 0.02
	
	

##  RESULTS

The number of participants was 1037, with 83% (*n* = 861) being mothers. Most participants were married (96%, *n* = 995), and the rest were either divorced or widowed (2% for each category). The participants were recruited from across five regions, involving 50 cities.

### Parents' Background Characteristics 

The participants' mean age was 39 ± 7.5 years and their educational level ranged from dropout (people who left school at the age of 16 years without formal degree) to Doctor of Philosophy (PhD) degree [Figure 1]. The participants' occupations were diverse – unemployed, civil employees, teachers, health professionals, security forces, entrepreneurs, and assistant executive officers. In terms of their monthly incomes, the responses ranged from under $1400 to > $8000 [Figure 2]. None of them had more than four children (one child: 564 [54%], two children: 407 [39%], three children: 75 [6%], four children: 15 [1%], respectively). Additionally, 18% (*n* = 187) had a general medical history (e.g., systemic hypertension, diabetes, asthma, thyroid gland dysfunction, and back pain). Lastly, approximately 33% of the participants reported some form of ocular disorder (e.g., refractive error, dry eye, cataract, keratoconus, amblyopia, and diabetic retinopathy).

### Parents' Eye Care-seeking Behavior

Interestingly, about half of the parents (467, 45%) responded that they had not taken their children to a vision screening at the entry of reception/year one. When asked if they were aware of the mandatory vision exam at the entry of reception/year one, 65% of the parents' responded with “NO” and another 13% with “maybe”. The parents' responses to questions directed toward the current visual status are summarized in Table 1. Regarding children who refused an eye test, only seven parents reported that they were given a reason for not being provided the service [Table 1]. Reasons included poor cooperation, young age, cost of service, waiting time, and presence of autism in a child.

The survey also checked to understand the reasons why parents would consider seeking eye care for their children. Their responses varied across different reasons as listed in Table 2. Some participants reported other reasons for intentionally seeking eye care, including excessive use of electronic devices, dry eyes, juvenile diabetes, eye redness, itching, and excessive blinking. In addition, the parents were surveyed based on their preexisting knowledge related to child vision and vision screening [Table 3].

### Barriers to Eye Care-seeking Behavior

The parents were asked about the barriers that might prevent them from taking their children for an eye test [Table 4]. Some parents mentioned additional barriers including their beliefs that the vision of their children was normal, the child being uncooperative, and personally not seeing a reason for an eye test and challenges with time management.

### Parents' Background Related to Their Knowledge and Barriers

An investigation was conducted to determine whether the responses were influenced by the parents' background or other related factors that included gender, marital status, age, income, working status, level of education, and family history of eye problems. After the investigation, it was ascertained that those characteristics influenced some of the parents' responses listed in Table 5.

##  DISCUSSION

This study showed that half of the parents had not taken their children for vision screening at reception/year one. The majority of them were not even aware of the mandatory screening program. Further, only one-fifth of them knew of the pathways to access eye-care services. About 60% of the children had not undergone any kind of eye or vision test. Barriers and misconceptions related to eye-care services, which needed intensive and in-depth strategies to deal with, were detected. Participants' backgrounds and socioeconomic characteristics also played a major role in some of the parents' responses.

Accessibility to vision screening is important for the well-being of children.^[10,40]^ Understanding the barriers to and knowledge of accessing eye-care services for children from a parental perspective is fundamental in determining strategies and programs that enrich the parents' awareness and provide methods to direct them for the best possible access to checking the vision of their children.^[32]^ Parental knowledge of risk factors related to not checking children's vision could contribute to early detection and management of various visual disorders, such as amblyopia and strabismus.^[34,35,41]^ This would also require the cooperation of eye-care professionals.^[7]^ In 2019, Cassetti et al suggested that it is imperative to consider parents' lack of eye health education as well as the importance of enhancing specialists' experience when treating children, and how to tackle parents' negative attitudes toward diagnosis and treatment.^[7]^

In this study, the percentage of parents whose children had received any kind of eye or vision test was closely similar to a report of a study in English children (45% vs 51%, respectively).^[32]^ In comparison to another study, our findings were better than those found in Swaziland children, where 60% of their participants had never taken their children for an eye test.^[38]^ Furthermore, concerns were raised about the efficacy of mandatory assessments at school reception/year one in light of poor awareness of the screening program, supported by the findings of Donaldson et al who reported that only 15% of the parents whose children go to a school with a screening program knew of its existence.^[32]^ Moreover, only a few participants had been given a reason for not being provided the service, and not allowing a child to undergo vision screening could cause major consequences on the child's well-being and quality of life.^[29,42]^ The reasons given by healthcare workers for not providing vision screening were mainly due to a lack of cooperation by the underaged subjects, a lack of financial resources by the parents, or the patients' ailments that would require more intricate testing and evaluation. Providing more professional training, giving out vouchers for eye examinations in schools, and easing the access to eye-care services provided by governmental hospitals may be very helpful in alleviating the lack of eye care for young children.^[7,32]^ In agreement with previous research, parents may also need more health education and more effective and accessible eye-care services.^[38,43,44]^

Parental misconceptions about eye examinations for children and their vision were the main barriers to taking children for a vision test. Similar to previous research, not knowing how and where to access eye-care services and not being able to afford the cost of service/glasses were other observed barriers.^[37,45,46]^

Effective efforts to correct those misconceptions, explaining the methods for accessing eye-care services and making these services free of charge are expected to increase the number of children taken to an eye test, exceeding the current 45% observed in this study. Potential strategies for enhancing parents' knowledge include distributing leaﬂets, providing online links to vision screening information, and giving out references to pathways for accessing eye-care services.^[32]^ In addition, it is important to advise parents that vision screening is not a comprehensive examination and it is only the first step as certain other conditions may be missed if complete examinations are not performed.^[32]^

The demographic and socioeconomic factors of the parents significantly influenced their responses, and this has also been supported in previous research.^[36]^ Based on the results reported in this study, educating new parents, easing accessibility to vision screening, making them free of charge, and increasing parental awareness across all working fields, including unemployed parents could increase the number of children being evaluated for vision.^[7,32]^ Although based on our findings, teachers could be important mediums to refer children for vision screening. Nevertheless, less educated parents and those with or without medical/ocular history should be properly educated about the importance of vision screening for children; education should focus more on providing fathers with more information about the vision of children and informing parents generally about patients' right to avoid/handle potential test refusal.^[18,37,38,47]^

Currently, no efficient national guidelines were applied to suggest pathways for vision screening, although the Ministry of Health and Education has recently agreed on a newer pathway for vision screening at school reception/year one and another at grade 4. However, the method in which the program would handle the referral for comprehensive eye examinations for children who fail the initial eye test is unclear, which may vary depending on local arrangements in different regions of SA as it was previously suggested in other countries.^[32]^ Moreover, studies have suggested that the most common reason for not undergoing comprehensive eye care after the child fails the initial vision screening was the parents' lack of knowledge about the outcome of the primary screening and/or what it means.^[18,47]^ Finally, Hartmann et al proposed developing a national integrated data system that would include child-level vision screening data, referral records, and follow-up diagnosis and treatment; following such a route can be very efficient.^[48]^

This study enrolled 1037 parents, most of whom were mothers (83%). The unbalanced gender recruitment could be a limitation of this study, although mothers may be more attached and closer to children than fathers. The recruited parents were diverse in terms of where they lived, their age, income, education, and the number of children. Furthermore, approximately one-third of the participants did experience some ocular disorders, indicating that they were aware of the importance of vision screening in children. This diversity in response could provide the representation required to reflect the assessment of the targeted population in different regions of the country. The recruitment method used in this study was not typical or similar to other studies that have distributed the questionnaires in hard copies in schools;^[32,33,45]^ however, the method in this study avoided possible bias that existed in other studies due to sample selection from a clinically based population.^[33]^ This could be because younger children may not have visited the eye clinic; alternatively, researchers might not have been able to distribute the questionnaires nationwide to have a sample representing the targeted population. That being said, the online survey may have some biases, like including the responses of parents who do not have any children under the age of seven, or parents who may ask for someone's help in responding to these inquiries, so it does not reflect their own thoughts and feelings about the topic. Although, we implanted a question in the survey to verify whether the respondents had children under seven years and excluded some of the collected data as stated in the method section, the responses of some parents who had no children under seven years of age might still have been included. Unfortunately, the second possible bias could not be verified, we were only able to trust the respondents' integrity and voluntary participation stated in their consent.

In summary, this study showed that the majority of parents lack the knowledge about the importance of vision screening and the existing pathways to accessing eye care for young children. It is recommended that parents' awareness of eye-care services be enhanced, and improved communication is needed to educate parents about the importance of vision screening for children, how to access eye-care services, and share knowledge of the existence of any national/mandatory screening programs. A second recommendation would be developing well-structured protocols to inform parents about their children's vision screening results and provide referral pathways to avoid any dropouts after failing school vision screening. And finally, a national protocol to cover the cost of eye services/glasses may be needed to address those parents who are unable to pay for the cost of eye services.

##  Financial Support and Sponsorship

None.

##  Conflicts of Interest

The author has no conflicts of interest to declare.
